# PLAG1 and NCAPG‐LCORL in livestock

**DOI:** 10.1111/asj.12417

**Published:** 2015-08-11

**Authors:** Akiko Takasuga

**Affiliations:** ^1^ National Livestock Breeding CenterFukushimaJapan

**Keywords:** body weight, height, NCAPG‐LCORL, PLAG1, selective sweep

## Abstract

A recent progress on stature genetics has revealed simple genetic architecture in livestock animals in contrast to that in humans. *PLAG1* and/or *NCAPG‐LCORL*, both of which are known as a locus for adult human height, have been detected for association with body weight/height in cattle and horses, and for selective sweep in dogs and pigs. The findings indicate a significant impact of these loci on mammalian growth or body size and usefulness of the natural variants for selective breeding. However, association with an unfavorable trait, such as late puberty or risk for a neuropathic disease, was also reported for the respective loci, indicating an importance to discriminate between causality and association. Here I review the recent findings on quantitative trait loci (QTL) for stature in livestock animals, mainly focusing on the *PLAG1* and *NCAPG‐LCORL* loci. I also describe our recent efforts to identify the causative variation for the third major locus for carcass weight in Japanese Black cattle.

## Introduction

Large‐scale genome‐wide association studies (GWAS) in humans have identified many novel loci for complex diseases and traits. Adult human height is a classical polygenic trait and has been analyzed as a model for quantitative genetics. Heritability of adult human height is estimated as approximately 80% (Fisher 1918; Silventoinen *et al*. 2003; Visscher *et al*. 2006), while only a small portion of the heritability was explained by the common variants identified by GWAS of tens of thousands of people (Visscher 2008; Manolio *et al*. 2009). To explain the “missing heritability”, a meta‐analysis combining several GWAS has been conducted to increase statistical power of detecting associated loci. The most recent GWAS for adult human height used the data from >253 000 individuals and identified 697 variants in 423 loci (Wood *et al*. 2014). The genome‐wide significant single nucleotide polymorphisms (SNPs) explained 16% of heritability and all common variants together captured 60% of heritability (Wood *et al*. 2014). The study also identified several genes and pathways not previously connected with human skeletal growth (Wood *et al*. 2014).

In livestock, quantitative trait locus (QTL) mapping and GWAS have been conducted for economically important traits. Several traits such as carcass weight, calving difficulty and puberty are known to relate with body size and have been extensively examined. We previously performed GWAS for carcass weight in Japanese Black cattle and revealed three major loci, designated as *CW‐1*, ‐*2* and ‐*3*, of which *CW‐1* and ‐*2* corresponded to adult human height loci, *PLAG1* and *NCAPG‐LCORL*, respectively (Setoguchi *et al*. 2009; Nishimura *et al*. 2012). The three loci together explained approximately one‐third of genetic variance of the GWAS population (Nishimura *et al*. 2012). The *PLAG1* and *NCAPG‐LCORL* regions were also detected for an association in different cattle breeds and other livestock species (Tables 1 and 2). The *CW‐3* QTL was detected in a specific line of Japanese Black (Nishimura *et al*. 2012), which hampered narrowing down the QTL region. However, recent advances in genomic technologies such as high‐density SNP chips and target resequencing enabled us to easily find a marker SNP in linkage disequilibrium (LD) with the QTL and screen candidate causative variations. The next challenge is to identify the causative variation. An excellent work by Karim *et al*. (2011), that identified quantitative trait nucleotides (QTNs) for bovine stature at the *PLAG1* locus, indicates that a comprehensive study with genetic, genomic and molecular biological approaches is required to identify the causative variation.

**Table 1:** Quantitative trait loci (QTLs) detected around the PLAG1 locus in livestock

Species	Breeds	No. of animals	Associated traits	Freq. of the height‐increasing allele	Reference
Cattle	F2 from Holstein‐Friesian and Jersey	864 cows	Height at 18 months of age, body weight at birth and 6, 12, 18 months of age, live weight	Holstein‐Friesian: 0.78; Jersey: 0.05	Karim *et al*. (2011)
	New Zealand Holstein‐Friesian	942 female calves	Peripubertal weight	0.85	Littlejohn *et al*. (2012)
	Japanese Black	1156 steers	Carcass weight	0.754	Nishimura *et al*. (2012)
	Japanese Black	768 steers	Peripubertal weight	0.79	Hoshiba *et al*. (2013)
	Brahman, Tropical Composite	Female cattle of 843 Brahman and 866 Tropical Composite	Age when first corpus luteum was observed, reduced blood insulin‐like growth factor (IGF)‐1 level at 24 months of age, hip height at 24 months of age, scanned rump fat thickness at P8 position	‐	Hawken *et al*. (2012)
	Brahman	1118 bulls	Blood IGF‐1 level at 6 months of age, Scrotal circumference at 12 months of age	‐	Fortes *et al*. (2012)
	Tropical Composite	1085 bulls	Blood IGF‐1 level at 6 months of age	‐	Fortes *et al*. (2013b)
	*Bos taurus*, Brahman, Tropical Composite	>1000 *Bos taurus* cattle	Live weight at feedlot entry, hip height at post‐weaning	*Bos taurus*: 0.96; Brahman: 0.5; Tropical Composite: 0.68	Fortes *et al*. (2013a)
		~1000 females of Brahman	Age when first corpus luteum was observed, reduced blood IGF‐1 level at the first wet season, live weight when first corpus luteum was observed		
		~1100 males of Brahman	Age at 26 cm of scrotal circumference		
		~1000 females of Tropical Composite	Age when first corpus luteum was observed, reduced blood IGF‐1 level at the first wet season, live weight when first corpus luteum was observed		
	Nellore	654 bulls	Estimated breeding value (EBV) for birth weight	0.18‐0.27	Utsunomiya *et al*. (2013)
	Nellore	861 bulls	EBV for scrotal circumference	MAF^a^ = 0.23	Utsunomiya *et al*. (2014)
	German Fleckvieh	1800 bulls	EBV for paternal calving ease, body size and daily gain	0.16	Pausch *et al*. (2011)
	US beef breeds	18 274 animals from 10 US beef breeds	Body weights, calving ease, carcass, weaning and yearling weights	‐	Saatchi *et al*. (2014)
Pig	European domestic pig populations, European wild boars, Asian domestic pigs	418 animals from European domestic pig populations, 40 European wild boars, 21 Asian domestic pigs	Candidate selective sweep region overlapped with a major QTL for body length in an intercross between Large White and wild boar	‐	Rubin *et al*. (2012)

a Minor allele frequency is shown in the study.

**Table 2:** Quantitative trait loci (QTLs) detected around the NCAPG‐LCORL locus in livestock

Species	Breeds	No. of animals	Associated traits	Freq. of the height‐increasing allele	Reference
Cattle	Japanese Black, Japanese Brown	>280 offspring × 5 half‐sib families	Carcass weight, longissimus muscle area, lower subcutaneous fat thickness	‐	Setoguchi *et al*. (2009)
	Japanese Black, F2 from Charolais and German Holstein	792 Japanese Black steers and 161 F2 bulls	Increase in body frame size at puberty	Japanease Black: 0.4; F2 bulls: 0.49	Setoguchi *et al*. (2011)
	Japanese Black	1156 steers	Carcass weight	0.2	Nishimura *et al*. (2012)
	F2 from Charolais and German Holstein	733 animals	Birth weight	Charolais: 0.9; German Holsteins: 0.2	Eberlein *et al*. (2009)
	F2 from Charolais and German Holstein	156 males	Increased plasma arginine level, average daily gain, lower percentage of perirenal and subcutaneous fat in carcass weight		Weikard *et al*. (2010)
	Crossbred	~2500 animals	Birth weight, weaning weight, yealing weight	‐	Snelling *et al*. (2010)
	Crossbred	400 animals (validation population)	Average feed intake, average daily gain, hot carcass weight, ribeye area, lower adjusted fat thickness	0.33	Lindholm‐Perry *et al*. (2011)
	Norwegian Red	2552 bulls	Daughter yield deviation for direct effect on dystocia	‐	Olsen *et al*. (2009)
	Piedmontese	323 bulls	Estimated breeding value for direct calving ease	‐	Bongiorni *et al*. (2012)
	US beef breeds	18 274 animals from 10 US beef breeds	Body weights, calving ease direct, weaning weight maternal	‐	Saatchi *et al*. (2014)
Horse	Franches‐Montagnes	1077 animals	Withers height, conformation of legs, ventral border of mandible, correctness of gait, expression of head	0.0845	Signer‐Hasler *et al*. (2012)
	German Warmblood	782 stallions	Withers height	0.65	Tetens *et al*. (2013)
	Hanoverian	214 animals	Height	0.55	Metzger *et al*. (2013)
	Thoroughbreds	282 affected and 268 controls	Height, laryngeal neuropathy	Affected: 0.259; Unaffected: 0.119	Boyko *et al*. (2014)
Pig	European domestic pig populations, European wild boars, Asian domestic pigs	418 animals from European domestic pig populations, 40 European wild boars, 21 Asian domestic pigs	Candidate selective sweep region overlapped with a major QTL for body length in an intercross between Large White and wild boar	‐	Rubin *et al*. (2012)
Dog	46 diverse breeds	509 animals	Highly differentiated region across breeds	‐	Vaysse *et al*. (2011)

Here I review the studies that reported a QTL or association around the *CW‐1* and ‐*2* regions, and then discuss a strategy to identify the causative variation using *CW‐3* as an example.

## Plag1

*PLAG1* (pleiomorphic adenoma gene 1) is a proto‐oncogene encoding a zinc‐finger containing a transcription factor of which ectopic expression is crucial in the formation of pleomorphic adenomas of salivary glands (Kas *et al*. 1997). The region including *PLAG1* and some neighboring genes (HSA8q12.1) has been identified as one of the loci for adult human height both in European (Gudbjartsson *et al*. 2008; Lettre *et al*. 2008; Lango Allen *et al*. 2010; Wood *et al*. 2014) and Asian populations (Cho *et al*. 2009; Kim *et al*. 2010; Okada *et al*. 2010), although associated SNPs were different between populations.

In cattle, Karim *et al*. (2011) identified regulatory QTNs locating between *PLAG1* and *CHCHD7* for stature (weight and height) in the F2 population from Holstein‐Friesian and Jersey cattle. The QTNs, locating at 25 Mb on BTA14 (UMD3.1), are comprised of a repeat number variation of a (CCG)*n* trinucleotide repeat and an adjacent SNP, both of which were shown to influence bi‐directional promoter strength and affect binding of nuclear factors (Karim *et al*. 2011). Consistent with these results, increased gene expressions of *PLAG1*, *CHCHD7* and other neighboring genes were observed in tissues from 79 fetuses in a height‐increasing allele and/or genotype‐dependent manner (Karim *et al*. 2011). A splice‐site variant of *CHCHD7* occurring in a null allele was found to have no additional effects on height or weight, resulting in elimination of *CHCHD7* as the only causative gene (Karim *et al*. 2011). In contrast, *PLAG1* appears the most promising causative gene for the QTL, because *Plag1* knockout mice show dwarfism in the absence of other symptoms (Hensen *et al*. 2004), showing consistency with the result that the bovine variant enhancing the gene expression increased withers height.

Table 1 shows the studies that detected a QTL or association around the *PLAG1* locus in livestock. In Japanese Black cattle, we detected the strongest association for carcass weight in an 830‐kb interval containing *PLAG1* (Nishimura *et al*. 2012). Target resequencing revealed that the *Q* haplotype in Japanese Black shared only QTNs with the *Q* haplotype of the F1 sires reported by Karim *et al*. (2011), which eliminated other candidate causative variations in the 780‐kb critical region identified by Karim *et al*. (2011) (Nishimura *et al*. 2012).

Fortes *et al*. (2013a) showed that the height‐increasing allele of *PLAG1* constitutes a small haplotype block in *Bos taurus* cattle but a 20‐Mb long haplotype in Brahman (*Bos indicus*), suggesting that the allele was recently introgressed into Brahman from *Bos taurus* cattle and strongly selected. The height‐increasing allele in *Bos taurus* also increases height and weight in Brahman and Tropical Composites, and was associated with decreased serum insulin‐like growth factor 1 (IGF1) level (Hawken *et al*. 2012; Fortes *et al*. 2012, 2013a,2013b). Since IGF1 is a growth stimulator, the direction of the association appears apparently paradoxical (Fortes *et al*. 2013a). The height‐increasing allele was also associated with late puberty in Brahman that was defined by observation of the first corpus luteum in heifers and age at 26 cm of scrotal circumference (SC) in bulls (Fortes *et al*. 2013a). In Tropical Composites, the association with late puberty was detected in heifers (Fortes *et al*. 2013a) but not in bulls (Fortes *et al*. 2013b). Utsunomiya *et al*. (2014) also detected an association of the region containing *PLAG1* with estimated breeding value (EBV) for SC in Nellore (*Bos indicus*) bulls, although the direction of the association is not indicated. Currently there is no information about *PLAG1* for affecting timing of puberty. In a mouse model, *Plag1*‐knockout mice show dwarfism and reduced fertility: *Plag1* ^−/−^ males impregnate wild‐type females at a lower rate and *Plag1* ^−/−^ females show reduced litter size (Hensen *et al*. 2004). A recent human GWAS using >182 000 women identified 106 genomic loci for age at menarche, in which the *PLAG1* locus was not detected (Perry *et al*. 2014). It is possible that late puberty in Brahman is caused by a hitchhiking effect of the introgressed allele, that is, another variation in a nearby gene affecting puberty.

Pausch *et al*. (2011) identified two genomic regions (on BTA14 and BTA21) associated with EBV for paternal calving ease in German Fleckvieh bulls, one of which was a 1.58‐Mb interval including *PLAG1*. Because the QTL alleles lowering EBV for calving ease were associated with increased EBV for body size and daily gain (Pausch *et al*. 2011), the stature QTNs at the *PLAG1* locus is likely causative for both calving ease and body weight. Utsunomiya *et al*. (2013) detected association of the *PLAG1* region with birth weight in Nellore (*Bos indicus*), although they did not show whether the *PLAG1* allele is segregated in the population. Recent human GWAS using tens of thousands of people identified several loci for birth weight (Horikoshi *et al*. 2013) and length (van der Valk *et al*. 2014), both of which included the adult human height loci, *HMGA2* and *LCORL*. Since HMGA2 stimulates *PLAG1* gene expression (Klemke *et al*. 2014), *PLAG1* might be another locus for birth weight and length in humans that was not detected in the GWAS.

Table 1 shows a frequency of the height‐increasing allele of the SNP with the strongest association in each study population. The frequencies are polarized in *Bos taurus* breeds: high in Holstein‐Friesian and Japanese Black, and low in Jersey and Fleckvieh.

In domestic pigs, Rubin *et al*. (2012) detected strong signature of selection in three regions, *NR6A1*, *PLAG1* and *LCORL*. Non‐synonymous amino acid substitution Pro192Leu in NR6A1 was shown to enhance binding to its co‐repressors and proposed to be causative to increase the numbers of vertebrae (Mikawa *et al*. 2007), while any obvious candidate mutations were not found in the coding sequences of *PLAG1* and *LCORL*, suggesting that regulatory mutations were selected for these genes (Rubin *et al*. 2012).

IGF2 and other growth factors have been identified as putative targets of PLAG1 (Voz *et al*. 2004). Recent studies showed that PLAG1 expression is stimulated by HMGA2 (Klemke *et al*. 2014) and decreased by miR‐141 (Tang *et al*. 2013). Future studies will reveal a detailed molecular network of PLAG1 to enhance skeletal growth, which will give a cue to understand apparently paradoxical associations between an increase in height/weight and a reduced serum IGF1 level or late puberty.

## NCAPG‐LCORL

The *NCAPG‐LCORL* region has been identified as a locus for adult human height in European (Gudbjartsson *et al*. 2008; Weedon *et al*. 2008; Soranzo *et al*. 2009; Lango Allen *et al*. 2010; Wood *et al*. 2014), Japanese (Okada *et al*. 2010) and African/African American populations (N'Diaye *et al*. 2011; Carty *et al*. 2012). The region is also associated with peak height velocity in infancy (PHV1) (Sovio *et al*. 2009), birth weight (Horikoshi *et al*. 2013) and birth length (van der Valk *et al*. 2014). The SNP with the strongest association locates in an intron of *LCORL* in European and African/African American populations but downstream of *DCAF16* in a Japanese population. N'Diaye *et al*. (2011) indicated that an LD block in a European population contains *DCAF16*, *NCAPG* and *LCORL*, while only *LCORL* is contained in an LD block of the individuals of African ancestry. Furthermore, the best associated SNP in African‐derived populations was associated with *LCORL* gene expression levels in lymphoblastoid cell lines derived from 56 unrelated Yoruba individuals (*P* = 0.0026) (N'Diaye *et al*. 2011). In contrast, Lango Allen *et al*. (2010) performed expression QTL (eQTL) analyses in lymphocyte (*n* = 830), osteoblast (*n* = 104), liver (*n* = 567) and omentum (*n* = 742) from individuals of European ancestry, in which lymphocyte and omentum eQTLs were found in this region but not correlated with the height SNP. Wood *et al*. (2014) also examined associations of the 697 height SNPs with eQTL in peripheral blood that was analyzed using the data from 2360 unrelated individuals of European ancestry and with non‐synonymous variants detected in the 1000 Genomes Phase 1 release. Neither eQTLs nor non‐synonymous variants were detected for association with the height SNP in this region.

There is no information indicating a link between height and a gene in this region. LCORL (ligand dependent nuclear receptor corepressor‐like) is a transcription factor that may function during spermatogenesis in the testes (Kunieda *et al*. 2003). NCAPG (non‐SMC condensin I complex, subunit G) is a regulatory subunit of the mammalian condensin I complex and is important during mitotic cell division (Dej *et al*. 2004). DCAF16 (DDB1 and CUL4 associated factor 16) may function as a substrate receptor for CUL4‐DDB1 E3 ubiquitin‐protein ligase complex (Wen *et al*. 2007). The LD analysis by N'Diaye *et al*. (2011) indicates that the causative variation should exist within *LCORL* at least in African‐derived populations, while the causative gene remains unknown. A recent study showed that obesity‐associated variants within introns of *FTO* are functionally connected, at megabase distances, with the homeobox gene *IRX3* (Smemo *et al*. 2014). In this locus, 3ʹ untranslated regions (UTRs) of *LCORL* and *NCAPG* are overlapped in human and mouse genomes (http://genome.ucsc.edu/cgi‐bin/hgGateway), raising the possibility of regulated alternate expression (http://www.ncbi.nlm.nih.gov/IEB/Research/Acembly/). Creation of *Lcorl*‐knockout mice may show direct involvement of *LCORL* in body length.

Table 2 shows the studies that detected a QTL or association around the *NCAPG‐LCORL* region in livestock. In cattle, the *NCAPG‐LCORL* region was associated with carcass weight (Setoguchi *et al*. 2009), birth weight (Eberlein *et al*. 2009; Snelling *et al*. 2010), weaning and yealing weight (Snelling *et al*. 2010), peri‐pubertal weight gain (Weikard *et al*. 2010) and increase in body frame size (Setoguchi *et al*. 2011). The LD block in Japanese Black encompassed a 591‐kb interval including *FAM184B*, *DCAF16*, *NCAPG* and *LCORL* and non‐synonymous amino acid substitution Ile442Met (pI442M) in NCAPG was found as a candidate causative variation (Setoguchi *et al*. 2009). The NCAPG pI442M allele was shared in the different cattle breeds used in these studies. Gutiérrez‐Gil *et al*. (2012) detected a bone QTL close to the *NCAPG‐LCORL* region, but excluded NCAPG pI442M (at 38.8 Mb on BTA6, UMD3.1) as a causal mutation and detected the strongest association with the SNP at 45.9 Mb on BTA6 (UMD3.1). In our analysis, one of the Japanese Black sires segregating the carcass weight QTL was homozygous between 39.2 and 61.0 Mb on BTA6 (UMD3.1), therefore the region was excluded (Setoguchi *et al*. 2009).

Interestingly, the *NCAPG‐LCORL* locus shows pleiotropy. Association of the height/weight‐increasing allele with reduced subcutaneous fat thickness has been consistently detected among the studies (Setoguchi *et al*. 2009; Weikard *et al*. 2010; Lindholm‐Perry *et al*. 2011; Hoshiba *et al*. 2013).

Association with calving ease or dystocia has been also detected around this region in different cattle breeds (Olsen *et al*. 2009; Bongiorni *et al*. 2012; Saatchi *et al*. 2014). The association may be explained by an increase in birth weight and length due to the height‐increasing allele, although the authors mentioned other candidate genes.

The region including *NCAPG‐LCORL* has been detected for association with height in several horse breeds (Signer‐Hasler *et al*. 2012; Tetens *et al*. 2013; Metzger *et al*. 2013; Boyko *et al*. 2014). Boyko *et al*. (2014) reported that the *LCORL* region was also associated with recurrent laryngeal neuropathy (RLN) explaining 6% of the variation. The height‐increasing allele was the risk allele for the disease, consistent with the clinically reported connection between height and RLN (Boyko *et al*. 2014).

The *LCORL* region was also identified as highly differentiated between dog breeds (Vaysse *et al*. 2011) and a selective sweep region in European domestic pigs (Rubin *et al*. 2012).

Association between gene expression levels of the candidate genes and genotypes or phenotypes has been examined in some studies. Eberlein *et al*. (2009) showed a trend that *NCAPG* gene expression was decreased according to numbers of the height‐increasing allele in fetal placenta, using three *qq*, two *Qq* and one *QQ* purebred German Holsteins: the *Q* denotes the height‐increasing allele encoding Met‐442 in NCAPG. Lindholm‐Perry *et al*. (2013) reported a negative correlation between average feed intake and *LCORL* transcript abundance in adipose tissue from cows (*n* = 81, *P* = 0.02) and heifers (*n* = 94, *P* = 0.045), while a positive correlation was detected between average feed intake and *LCORL* transcript abundance (*P* = 0.04) or protein level (*P* = 0.01) in muscle tissues from steers (*n* = 31). In muscle tissues from cows (*n* = 86), *NCAPG* transcript abundance was associated with average daily gain (*P* = 0.009) (Lindholm‐Perry *et al*. 2013). Weikard *et al*. (2010) conducted metabolic profiling (*n* = 156 male cattle) to reveal a physiological pathway related with this locus and showed that the amino acid arginine and its metabolite symmetric dimethylarginine were associated with the height‐increasing allele (NCAPG pI442M). Metzger *et al*. (2013) reported that a variant locating at 62 kb‐upstream region of *LCORL*, which is in a transcription factor II D (TFIID)‐binding motif, was associated with horse height and that the height‐increasing allele was associated with reduced gene expression of *LCORL* in hair root (total 44 horses from five breeds).

## Genetic architecture for stature

Each locus for adult human height explains ~0.3% to ~0.5% of the phenotypic variance (Visscher 2008), corresponding to less than 0.625% of genetic variation (calculated by assuming heritability as 80%). The genome‐wide significant SNPs in 423 loci together explained 16% of heritability (Wood *et al*. 2014). In contrast, genetic architecture for stature is simple in domestic animals. In cattle, several major loci including *PLAG1* and *NCAPG‐LCORL* explained more than a third of genetic variance in a breed (Saatchi *et al*. 2014). In horses, only the *LCORL* region explained 11% and 18% of the phenotypic variance in Franches‐Montagnes and German Warmblood, respectively (Signer‐Hasler *et al*. 2012; Tetens *et al*. 2013). Makvandi‐Nejad *et al*. (2012) reported that four loci explained 83% of size variance in the 48 horses from 16 breeds and two loci explained 59% of the variance in thoroughbred size, although these estimates are likely to be upwardly biased by the small and selected sample. The two loci, *PLAG1* and *LCORL*, together explained 18.4% of the residual variance in body length in an intercross between Large White pigs and wild boar (Rubin *et al*. 2012).

A locus with a large effect on a favorable trait tends to be strongly selected and fixed in domestic animals. Identification of the causal variants may reveal why the effect size is large in domestic animals. Human height loci are substantially enriched for regulatory variants (Wood *et al*. 2014). The bovine QTNs at the *PLAG1* locus are regulatory and increased less than two‐fold gene expression (average 1.2‐fold), while the QTL genotype effects on live weight were +19.9 kg (*QQ*), 0 kg (*Qq*) and −23.5 kg (*qq*) and the QTL explained 9.9% of live weight variance of the F2 population (Karim *et al*. 2011). The large effect may be explained by a critical role of the PLAG1 transcription factor in up‐regulating growth factors (Voz *et al*. 2004).

### CW‐3

The *CW‐3* QTL on BTA8 have been detected in several paternal half‐sib families in Japanese Black the sires of which shared an identical‐by‐descendant haplotype encompassing a >14 Mb interval (Nishimura *et al*. 2012). Imputation of the 50K genotypes from the GWAS to high‐density SNP genotypes and a haplotype‐based association analysis highlighted a 3.3 Mb interval as a candidate region (Takasuga A, 2014, unpublished data). Below I describe the efforts that have been made to identify the causative variation and pitfalls of the respective analyses.

1. Target resequencing: many candidate causative variations were obtained, while GC‐rich and repeat regions remained uncovered. For example, the causative variations at the *PLAG1* locus were located within a GC‐rich region and were not covered by target resequencing (Nishimura *et al*. 2012).
2. Expression analysis: it is usually difficult to obtain tissue samples of livestock animals in an appropriate growth stage and with an appropriate genotype. A database search for human (http://www.genecards.org/) and mouse gene expression (http://www.informatics.jax.org/expression.shtml) may be helpful, although some differences may exist among species. A candidate gene for *CW‐3* was expressed specifically in a growth plate from a Holstein femur, while the mouse ortholog was broadly expressed in a femur (Takasuga A, 2014, unpublished data).
3. Literature analysis to select functional candidates: OMIM (http://www.omim.org/) and a catalog of published genome‐wide association studies (http://www.genome.gov/gwastudies/) may be helpful to select a primary candidate. In the case of *CW‐3*, the 3.3 Mb candidate region included several genes that are involved in skeletal growth and/or associated with adult human height (Nishimura *et al*. 2012).
4. *In vivo* studies using a knockout mouse: International Mouse Strain Resource (http://www.findmice.org/) provides mutant mouse and ES cell lines. Taconic Biosciences (http://www.taconic.com/) is a commercial supplier that assorts more than 4000 knockout mouse lines in the repository. We found a non‐synonymous variation in a gene associated with adult human height, which was predicted to be a damaging mutation by SIFT (http://sift.bii.a‐star.edu.sg/) and Polyphen‐2 (http://genetics.bwh.harvard.edu/pph2/). But the mice deficient for the gene showed neither abnormalities nor a change in body weight and length (Takasuga A, 2014, unpublished data). The lesson from the study is that a damaging mutation can be present in a gene that is not essential for growth.

Recently we detected a linkage between *CW‐3* and the skeletal abnormalities characterized by joint‐ and/or hip bone‐enlargement (Takasuga A, 2014, unpublished data). Now we are pursuing the causal mutation for the genetic disease, because the causal mutation is probably not a regulatory mutation but a non‐synonymous or a splicing‐junction mutation altering a coding sequence. Since disease mutations are usually infrequent, they may be distinguished from common variants using the data from whole‐genome sequencing of 234 bulls from various breeds (Daetwyler *et al*. 2014) and of pooled libraries from 54 steers in Japanese Black (Hirano *et al*. 2013).

#### Perspectives

In livestock, genomic loci with a large effect on a favorable trait have been often accompanied with an unfavorable character. Non‐synonymous variant Lys232Ala in DGAT1 increases fat content but decreases milk yield in cattle (Grisart *et al*. 2001, 2004). A frame‐shift or Cys636Arg mutation in MRC2 increases muscularity in a heterozygous carrier status but causes the recessive Crooked Tail Syndrome in Belgian Blue cattle (Fasquelle *et al*. 2009; Sartelet *et al*. 2012). Therefore it is important to identify whether the unfavorable character (late puberty in Brahman cattle, RLN in horses and the skeletal abnormalities in Japanese Black cattle) is caused by the causative variation for stature or by a closely located but different mutation. Although many efforts with *in vitro* and *in vivo* studies are needed to identify the causative variation, they cannot be avoided. A large effect size may make it easier to identify the causative variation in domestic animals than in human, and in turn, identified causative variation may explain its large effect size and will give an insight to understand the underlying molecular mechanism.
